# Relation between socioeconomic status and maternal serum lipids to infant lipid concentrations and anthropometry in the first year of life

**DOI:** 10.1007/s00404-023-06937-6

**Published:** 2023-03-02

**Authors:** Anne Dathan-Stumpf, Mandy Vogel, Nico Grafe, Wieland Kiess, Holger Stepan

**Affiliations:** 1 Department of Obstetrics, University Hospital Leipzig, Liebigstraße 20a, 04103 Leipzig, Germany; 2 LIFE Leipzig Research Center for Civilization Diseases, University of Leipzig, 04103 Leipzig, Germany; 3 Department of Pediatrics, University Hospital Leipzig, 04103 Leipzig, Germany

**Keywords:** Serum lipids, Pregnancy, Socioeconomic status, Winkler Index, LIFE Child, Anthropometry

## Abstract

**Purpose:**

The physical health and development of an individual are influenced by multiple parameters and shaped by internal and external factors during pregnancy. However, it is unclear whether there is an association between maternal lipid concentrations in the third trimester of pregnancy and infant serum lipids as well as anthropometric growth, and whether these factors are influenced by the socioeconomic status (SES) of the mothers.

**Methods:**

Between 2011 and 2021, 982 mother–child pairs were recruited in the LIFE-Child study. To investigate the influence of prenatal factors, pregnant women at the 24th and 36th week of gestation as well as children at the age of 3, 6 and 12 months were examined and serum lipids determined. Socioeconomic status (SES) was assessed using the validated Winkler Index.

**Results:**

A higher maternal BMI was associated with a significantly lower Winkler score and a higher infant weight, height, head circumference and BMI from birth up to the 4th–5th week of life. In addition, the Winkler Index correlates with maternal HDL cholesterol and ApoA1 levels. There was no relation between the delivery mode and the maternal BMI or SES.

For the maternal HDL cholesterol concentration in the third trimester, an inverse relation to children’s height, weight, head circumference and BMI up to the first year of life as well as the chest and abdominal circumference to an age of 3 months was found. Children born to mothers with dyslipidemia in pregnancy tended to have a worse lipid profile than those born to normolipidemic mothers.

**Conclusion:**

Serum lipid concentrations and anthropometric parameters of children in the first year of life are affected by multiple factors like maternal BMI, lipid levels and SES.

## What does this study add to the clinical work

**Table:** 

The results show first in an exemplary manner the complex, epigenetic interactions on children’s lipids and growth in a German mother–child cohort.	

## Introduction

The physical health and development of an individual are influenced by multiple parameters and shaped by internal and external factors during pregnancy. As already shown with the LIFE Child (Pregnancy) cohort, the serum lipids show strong physiological concentration changes during pregnancy, whereby these do not correlate with dietary habits, food intake or parity [1]. However, it is questionable if these changes in concentration are also influenced by socioeconomic factors, like school education, net household income and wealth of the mother.

In the literature, the Winkler Index is considered a valid measurement to categorize socioeconomic status (SES) by index scores [2]. The Winkler Index correlates with the serum lipid concentrations in childhood, a lower SES is associated with significantly more adverse lipid profiles [3]. It is unclear, how the maternal concentration of serum lipids in the third trimester affects the lipid levels in their newborn, especially within the first year of life. In addition, socioeconomic factors also seem to influence children’s growth. A lower maternal SES is associated with lower birth weight and preterm birth [4, 5].

Studies have shown that high maternal triglycerides and low high-density lipoprotein (HDL) levels are associated with fetal macrosomia, regardless of the presence of a diabetic metabolic state [6–9]. However, whether lipid metabolism also influences anthropometric variables such as head and waist circumference from birth to the first year of life cannot be determined from the current literature.

Therefore, the aim of this study is to compare the serum lipid concentrations of pregnant women with those of newborns up to the age of at least 12 months in a large German mother–child cohort. In addition, the influence of socioeconomic status on maternal lipid concentrations in the third trimester and the children growth within the first year of life is examined.

## Materials and methods

### Study population and design

This study is based on the LIFE Child study, which aims to detect and analyze environmental factors related to the growth, development and health of newborns, children and adolescents. LIFE Child is an ongoing longitudinal study based in Leipzig (Germany). To investigate the influence of prenatal factors, pregnant women between the 24th and 36th week of gestation (wog) (+ two weeks) as well as children aged between 0 and 18 years with yearly follow-up visits were recruited. In the first year of life, additional study examinations are carried out at the age of 3, 6 and 12 months. The study program includes—amongst others—anthropometry measurements, clinical exams, questionnaires as well as the assessment of biological samples (blood, urine, mother milk, hair) [10, 11]. Also as part of the LIFE Child study, blood pressure is measured after a 5-min rest period in one-minute measurement intervals with a cuff size adapted to the body constitution. A total of three values are measured. Flyers and notices about the participation in the LIFE Child study can be found in the obstetric outpatient clinics in Leipzig as well as pediatric practices. The study collective is primarily of Caucasian origin.

Between 2011 and 2021, 982 mother–child pairs were recruited. Information on the mode of delivery, the child's birth measurements and outcome parameters were taken from the maternity record and the yellow U-booklets at the first study visit. Likewise, all anthropometric measurement data of the U-examinations are taken from the yellow booklets and updated during the annual visits.

Patients on lipid-lowering medication were excluded from analyses, but no one fulfilled this criterion. The LIFE-Child cohort is a healthy collective [1].

The study was approved by the Ethical Committee of the University of Leipzig (reference number: Reg. No. 264-10-19042010). LIFE Child is registered by the trial number: NCT02550236.

### U-Examination

In Germany, a nationwide uniform early detection program for children from birth to school age exists, which gives every child a legal right to ten free early detection examinations—the U1–U9. With each examination, there is a questioning or updating of the personal, medication, illness and social anamnesis. In addition to motoric and sensory development examinations, physical inspection and general examinations, a standardized measurement of weight measured by a calibrated scale, height and head circumference is implemented. The results are documented in the yellow U-booklets. The standardized U-examinations take place at the following times: U1: immediately after the birth, U2: 3rd–10th day of life, U3: 4th–5th week of life, U4: 3rd–4th month of life, U5: 6th–7th month of life, U6: 10th–12th life month. Annual check-ups (U7, U7a, U8, U9) take place up to the 5th birthday.

### Lipid measurements

Venous blood was taken from the fasting subjects of the LIFE study. The predetermined fasting times were 12 h in the second and at least four hours in the third trimester because prolonged fasting periods did not seem acceptable. Fasting times less than 9–12 h may affect the level of triglycerides especially. Non-compliance was not an exclusion criterion, but most subjects adhered to the predetermination. Non-compliance was observed only in very few cases in the second trimester. Since in the following, we will primarily focus on the correlation for triglyceride concentrations in the 36th week of gestation, the different fasting times in the second and third trimesters are of limited importance. There are no prescribed fasting times for children up to the U7 examination.

Due to the fact that many parents refuse invasive blood sampling in infancy, there are a large number of missing values. Therefore, a small number of mother–child pairs with complete lipid profiles result.

The measurement of laboratory parameters was carried out in the Institute for Laboratory Medicine of the University Hospital. The measurement of serum lipids was performed on a ‘Cobas 8000 Clinical Chemistry Analyzer’ with test kits from Roche Diagnostics GmbH. The determination of total cholesterol, HDL cholesterol, LDL cholesterol and triglycerides was performed using a validated specific homozygous enzymatic color test. LDL cholesterol was also measured and not calculated according to Friedewald. ApoA1 and ApoB were determined by immunological turbidity testing. Non-HDL cholesterol was calculated from the difference between total cholesterol and HDL cholesterol. As dyslipidemia a value ≤ 5th percentile in HDL cholesterol and ApoA1 as well as a value ≥ 95th percentile in total cholesterol, triglycerides, LDL cholesterol or ApoB of reference value in pregnant women was defined [1].

### Socioeconomic status

The Winkler Index, which is made up of the three characteristics education, professional position and net income, was used to operationalize the socioeconomic status (SES). The index is calculated additively as an unweighted total score and can be treated as a metric variable in statistical analyzes even if the sub-dimensions only have an ordinal scale level [12]. From the total score, which can have values between 3 and 21, a categorization into social classes can be made. For each child, a value for both, mother and father, was determined and an overall score was derived. A score of 3–8 points is considered as low, 9–14 points as medium and 15–21 points as high SES. The Winkler Index was originally developed for the 1998 Federal Health Survey [13] and was validated and used in various studies, including the KiGGS [14]. The point value of the Winkler Index in the 36th wog was used for the correlation and regression analyses.

### Statistical analysis

The statistical analysis was carried out with the IBM Statistical Package for Social Sciences (IBM SPSS V.27). Plausibility for all values was tested. Missing values, for example, due to non-attendance to the yearly study visit, were handled as Missing Completely at Random. Standardized statistical methods were used. For testing the relation between delivery modes and presentation the Chi-square test as well as the *z* test were used. The Wilcoxon–Mann–Whitney *U* test and the Kruskal–Wallis test were used as non-parametric tests of independent samples. To adjust one or more independent variables, the conventional correlation analyzes were supplemented by multivariate regressions. For the multivariate regression analyses, the Winkler score and the maternal BMI value were chosen as independent and the maternal lipid parameters as dependent variables, since these showed significant interdependencies in the correlation, respectively, univariate regression analyses. A value of < 5% was used as the level of significance.

In Table 4 and S4, only significant results for the correlational analyses were shown.

**Table 1:** Description of the total study collective as well as according to the gestational age around the 24th and 36th week of pregnancy for maternal age, body-mass-index and levels of serum lipids

		*N*	Mean	min	max	95% CI		SD
BMI [kg/m^2^]	Total		26.90	17.62	55.64	26.65	27.16	4.74
	24 wog	839	26.33	17.62	55.64	26.01	26.65	4.75
	36 wog	386	28.26	20.17	48.00	27.81	28.72	4.55
Age [years]	Total		31.16	18.51	55.27	30.96	31.36	4.38
	24 wog	857	30.91	18.51	55.27	30.61	31.21	4.43
	36 wog	894	31.20	18.75	46.21	30.92	31.48	4.32
Cholesterol [mmol/l]	Total		6.56	3.17	13.25	6.51	6.62	1.24
	24 wog	840	6.21	3.17	12.12	6.13	6.28	1.12
	36 wog	870	6.92	3.71	13.25	6.83	7.00	1.25
HDL cholesterol [mmol/l]	Total		2.06	0.90	4.26	2.04	2.08	0.46
	24 wog	840	2.14	0.90	3.70	2.11	2.17	0.46
	36 wog	870	2.00	0.90	4.26	1.97	2.03	0.47
LDL cholesterol [mmol/l]	Total		4.11	1.24	9.56	4.03	4.19	1.15
	24 wog	397	3.80	1.24	8.74	3.69	3.90	1.03
	36 wog	415	4.39	1.25	9.56	4.28	4.50	1.17
Triglycerides [mmol/l]	Total		2.20	0.56	8.70	2.16	2.24	0.89
	24 wog	838	1.74	0.76	5.02	1.70	1.78	0.59
	36 wog	870	2.64	0.56	8.70	2.58	2.70	0.90
ApoA1 [g/l]	Total		2.18	1.21	3.33	2.15	2.20	0.33
	24 wog	351	2.16	1.21	3.10	2.13	2.20	0.32
	36 wog	356	2.20	1.25	3.33	2.16	2.24	0.35
ApoB [g/l]	Total		1.33	0.47	2.99	1.31	1.36	0.35
	24 wog	350	1.22	0.47	2.60	1.19	1.26	0.32
	36 wog	356	1.45	0.50	2.99	1.41	1.49	0.36
Non-HDL cholesterol [mmol/l]	Total		4.50	1.51	10.81	4.44	4.55	1.26
	24 wog	840	4.06	1.51	9.63	3.99	4.13	1.09
	36 wog	870	4.93	1.79	10.81	4.85	5.01	1.27

*N* number, *min* minimum, *max* maximum, *95% CI* 95% confidence interval, *SD* standard deviation, *BMI* body-mass-index, *HDL* high-density lipoprotein, *LDL* low-density lipoprotein, *wog* weeks of gestation

For correlation analyzes, the Standard Deviation Score (SDS) values of children's anthropometric parameters and blood pressure values, which are based on the reference values of the KIGGS study of the federal health report [15], were used.

## Results

### Study cohort

982 mother–child pairs were included in the study, 857 in the second trimester (24th week of gestation) and 894 in the third trimester (36th week of gestation). The mean maternal age was 31.2 years (Table 1), and the mean gestational age at delivery was 39.3 wog. The characteristics of the study collective are given in Supplement, Table S1.

**Table 2:** Representation of various anthropometric measurements from children at the respective examination times and their relation to the socioeconomic status (SES) of their families as measured by the Winkler Index, analyzed by Spearman correlation

	Age	*N*	Mean	min	max	95% CI		SD	SES	
									*r*	*p* value
**Anthropometry**										
Height [cm]	3 months	839	60.6	50.5	73.0	60.42	60.79	2.77	0.04	0.30
	6 months	834	67.5	58.0	75.5	67.34	67.72	2.77	0.04	0.24
	12 months	741	75.4	66.5	89.5	75.23	75.65	2.90	− 0.06	0.11
Weight [kg]	3 months	838	5.8	3.4	8.4	5.75	5.87	0.83	0.04	0.30
	6 months	835	7.5	4.8	11.7	7.45	7.58	0.98	0.03	0.33
	12 months	748	9.4	6.5	13.3	9.30	9.45	1.06	− 0.05	0.22
BMI [kg/m^2^]	3 months	836	15.8	12.1	21.5	15.67	15.87	1.48	0.03	0.45
	6 months	834	16.4	12.1	21.4	16.34	16.53	1.40	0.01	0.71
	12 months	741	16.4	12.7	20.7	16.35	16.53	1.24	− 0.01	0.81
Head girth [cm]	3 months	840	39.8	35.6	44.7	39.70	39.90	1.42	0.06	0.09
	6 months	833	42.8	38.7	51.0	42.72	42.92	1.48	0.06	0.08
	12 months	736	45.7	35.9	55.1	45.59	45.82	1.63	0.03	0.36
Biparietal length [cm]	3 months	658	22.6	18.5	27.0	22.46	22.66	1.26	0.00	0.95
	6 months	645	24.8	21.4	43.8	24.64	24.91	1.70	− 0.01	0.78
	12 months	521	26.2	22.7	46.5	26.08	26.38	1.77	0.02	0.58
Chest girth [cm]	3 months	841	39.7	32.8	47.2	39.53	39.82	2.19	0.07	**0.05**
	6 months	832	42.8	26.9	58.0	42.68	43.01	2.36	0.02	0.49
	12 months	725	45.7	36.8	53.0	45.51	45.82	2.13	− 0.02	0.54
Abdominal girth [cm]	3 months	839	38.9	29.0	47.9	38.72	39.11	2.88	0.07	**0.03**
	6 months	832	41.2	33.6	52.2	41.00	41.38	2.84	0.04	0.26
	12 months	717	43.3	31.1	54.4	43.08	43.51	2.97	0.02	0.65

For the correlation analysis, the SDS-values of anthropometry measurements were used. A *p* value < 0.05 was considered as significant. Significant associations are highlighted in bold

*N* number, *min* minimum, *max* maximum, *95% CI* 95% confidence interval, *SD* standard deviation, *SES* Socioeconomic status as measured by the Winkler Index, *r* correlation by Spearman, *SDS* standard deviation score

### Socioeconomic status

Measured by the Winkler Index, only 3.6% (*n* = 34) of the mother–child couples had a low SES. 529 (56.7%) could be assigned to a medium and 370 (39.7%) to a high SES. The mean value for the Winkler Index in the entire collective was 14.56 ± 0.11 points. With regard to the highest level of education and professional status, the mean scores of the two parents do not differ.

A higher maternal BMI was associated with a significantly lower Winkler score and thus, SES (*r* = – 0.19; *p* = 0.002). In addition, the Winkler Index correlated with the maternal HDL cholesterol level (*r* = 0.11; *p* = 0.005) and the ApoA1 concentration (*r* = 0.15; *p* = 0.013) in the 36th week of gestation. No influence of the SES on children’s serum lipid concentrations, blood pressure values or pulse frequency at the age of 3, 6 or 12 months could be demonstrated (Supplement, Table S4).

### Anthropometry

**Fig. 1:**
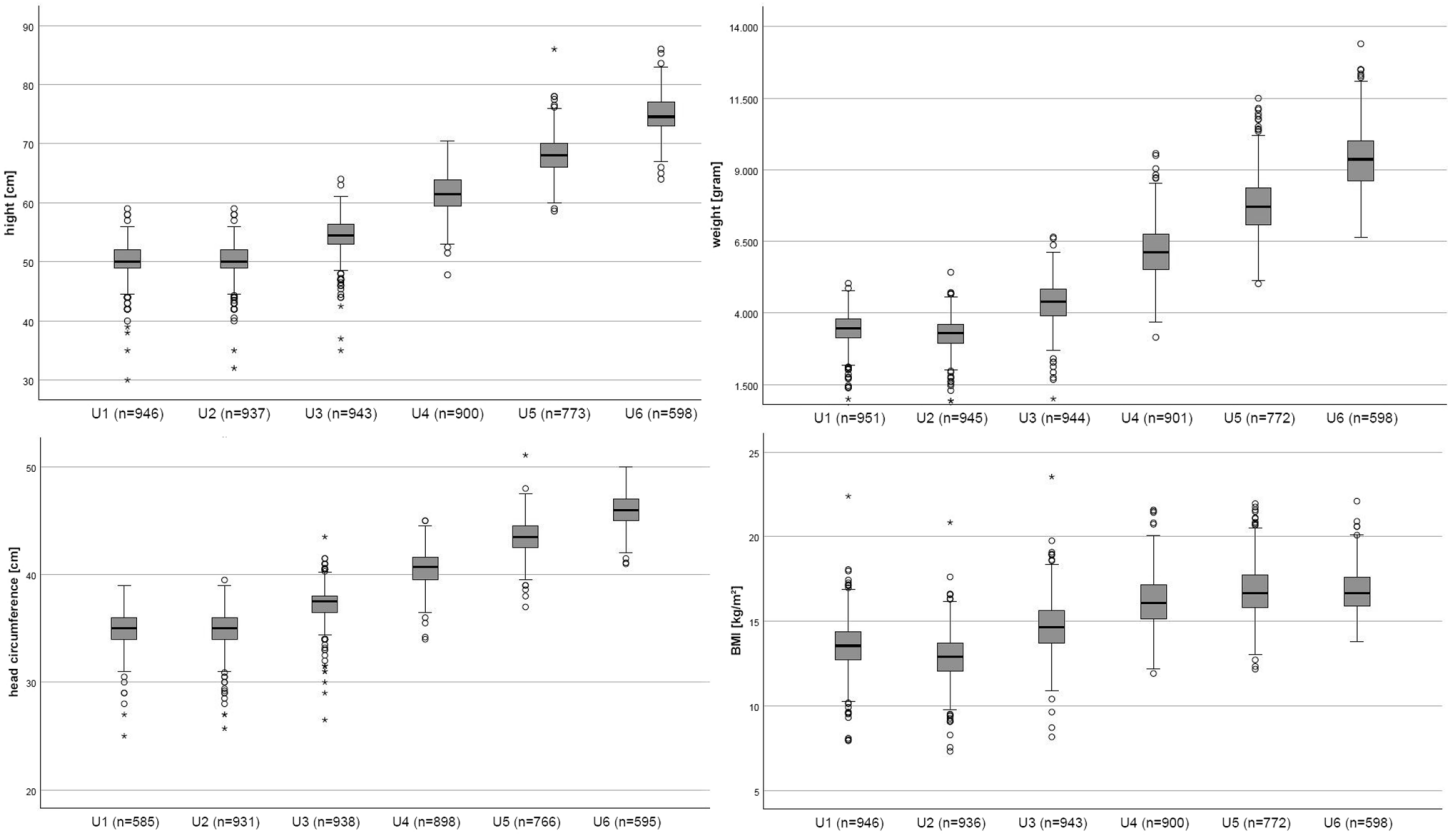
Development in terms of height, weight, head circumference and BMI from the U1 to U6 examination using box plots. The used box plots are conventional box whisker plots. In the box, the dash marks the median (50% quantile), the lower limit of the box is characterized as the first quantile and the upper limit as the third quantile. The crisscross represents the mean. The maximum length of the strokes up and down are 1.5 times of the interquartile ranges. The outliers are represented as points, extreme outliers as stars

The development of height, weight, head circumference and BMI for the respective U-examinations up to the U6 are shown in Fig. 1. The values of the anthropometric measurements at the respective U-examinations can be found in Supplement, Table S3. The blood pressure values and heart rates at the ages of 3, 6 and 12 months are also listed in Supplement, Table S4. Table 2 shows additional infantile anthropometric parameters at the age of 3, 6 and 12 months and their relation to the SES measured by the Winkler Index. Only the chest (*r* = 0.07; *p* = 0.047) and the abdominal girth (*r* = 0.07; *p* = 0.03) at the age of 3 months showed a significant correlation to the SES (Table 2).

**Table 3:** Relation between the maternal BMI and concentration of serum lipids at 36th weeks of gestation and various parameters of children’s anthropometry and lipid concentrations

Data child	At 36 weeks of pregnancy										
	Mat. BMI		HDL			Triglycerides		ApoA1		Non-HDL	
	*r*	*p*	*r*	*p*	*p*^*a*^	*r*	*p*	*r*	*p*	*r*	*p*
**U1**											
SDS-weight [gram]	0.25	< 0.001	– 0.24	< 0.001	0.045	0.15	0.001	– 0.27	< 0.001	0.14	0.004
SDS-hight [cm]	0.17	0.024	– 0.11	0.015				– 0.14	0.023		
SDS-head circ. [cm]			– 0.22	< 0.001		0.14	0.019	– 0.24	0.003		
SDS-BMI [kg/m^2^]	0.18	0.019	– 0.19	< 0.001		0.17	< 0.001	– 0.26	< 0.001	0.17	< 0.001
**U2**											
SDS-weight [gram]	0.24	< 0.001	– 0.24	< 0.001		0.15	0.001	– 0.31	< 0.001	0.12	0.010
SDS-hight [cm]	0.14	0.017	– 0.13	0.006							
SDS-head circ. [cm]	0.17	0.004	– 0.02	0.002		0.10	0.029	– 0.23	0.003		
SDS-BMI [kg/m^2^]	0.22	< 0.001	– 0.20	< 0.001		0.17	< 0.001	– 0.32	< 0.001	0.16	< 0.001
**U3**											
SDS-weight [gram]	0.21	< 0.001	– 0.22	< 0.001		0.11	0.022	– 0.21	0.007		
SDS-hight [cm]	0.15	0.009	– 0.14	0.003				– 0.15	0.060		
SDS-head circ. [cm]	0.13	0.028	– 0.12	0.011				– 0.25	0.002		
SDS-BMI [kg/m^2^]	0.13	0.023	– 0.19	< 0.001		0.15	0.001	– 0.24	0.003		
**U4**											
SDS-weight [gram]			– 0.15	0.002		0.13	0.008				
SDS-hight [cm]			– 0.09	0.018							
SDS-head circ. [cm]			– 0.10	0.029		0.11	0.040				
SDS-BMI [kg/m^2^]			– 0.14	0.004		0.15	0.002				
**U5**											
SDS-weight [gram]	0.13	0.044	– 0.09	0.037		0.10	0.014				
SDS-hight [cm]			– 0.11	0.006							
SDS-head circ. [cm]			– 0.12	0.004							
SDS-BMI [kg/m^2^]						0.09	0.037				
**U6**											
SDS-weight [gram]			– 0.11	0.022							
SDS-hight [cm]	0.15	0.041	– 0.10	0.030							
SDS-head circ. [cm]			– 0.12	0.010							
SDS-BMI [kg/m^2^]											
**3 months**											
SDS-weight [gram]			– 0.12	0.002		0.10	0.010				
SDS-head circ. [cm]			– 0.12	0.003	0.046			– 0.13	0.049		
SDS-chest girth [cm]			– 0.11	0.004		0.08	0.045				
SDS-abdominal girth [cm]			– 0.08	0.036		0.09	0.019				
**6 months**											
SDS-weight [gram]						0.12	0.001				
SDS-head circ. [cm]			– 0.09	0.022		0.09	0.018				
SDS-chest girth [cm]						0.10	0.006				
SDS-abdominal girth [cm]						0.11	0.004				
**12 months**											
SDS-weight [gram]	0.13	0.036	– 0.08	0.035		0.08	0.050				
SDS-head circ. [cm]								– 0.13	0.030		
SDS-chest girth [cm]											
SDS-abdominal girth [cm]											

Data child	At 36 weeks of gestation									
	Cholesterol		HDL		LDL		ApoA1		Apo B	
	*r*	*p*	*r*	*p*	*r*	*p*	*r*	*p*	*r*	*p*
**3 months**										
Total cholesterol [mmol/l]	0.25	0.006	0.30	< 0.001						
HDL cholesterol [mmol/l]			0.22	0.015			0.24	0.007		
LDL cholesterol [mmol/l]	0.22	0.016	0.23	0.001	0.18	0.049				
Triglycerides [mmol/l]										
ApoA1 [g/l]			0.22	0.023			0.22	0.026		
ApoB [g/l]	0.25	0.007			0.22	0.017				
Non-HDL [mmol/l]										
**6 months**										
Total cholesterol [mmol/l]	0.02	0.032	0.21	0.015						
HDL cholesterol [mmol/l]										
LDL cholesterol [mmol/l]										
Triglycerides [mmol/l]	0.18	0.045			0.21	0.018			0.19	0.033
ApoA1 [g/l]										
ApoB [g/l]										
Non-HDL [mmol/l]										
**12 month**s										
Total cholesterol [mmol/l]										
HDL cholesterol [mmol/l]			0.28	0.017			0.24	0.045		
LDL cholesterol [mmol/l]										
Triglycerides [mmol/l]										
ApoA1 [g/l]										
ApoB [g/l]					0.28	0.016			0.31	0.008
Non-HDL [mmol/l]										

For the Pearson correlation analysis, the SDS-values of anthropometry measurements were used. A *p* value < 0.05 was considered as significant. Only significant correlations are shown in the table. No significant relation could be found for adjusting the model by multivariate regression analysis for maternal BMI AND socioeconomic status. For the investigation period of U7–U8 (approximately 24–48 months of age) no more significant correlations were found

*r* correlation by Pearson, *p* *p* value, *Mat. BMI* maternal Body-Mass-Index, *HDL* High-Density Lipoprotein, *LDL* Low-Density Lipoprotein, *SDS* standard deviation score, *circ.* circumference

^a^Adjusted for maternal BMI

The maternal BMI in the 36th wog showed a strong positive correlation to the child's weight, height, head circumference and BMI from birth up to the U3 (4th–5th week of life). To note, for the following U-examinations this relation could no longer be shown (Table 3). Additionally, this correlation to infantile anthropometry can already be shown for the maternal BMI in the 24th week of gestation (Supplement, Table S5).

**Table 4:** Presentation of children's serum lipid levels at the age of 3, 6 and 12 months, divided according to maternal normolipidaemia and dyslipidaemia

	Age	Mother with non-dyslipidaemia							Mother with dyslipidaemia							*P*
		*N*	Mean	min	max	95% CI		SD	*N*	Mean	min	max	95% CI		SD	
**Serum lipids children**																
Total cholesterol [mmol/l]	3 months	76	3.86	2.30	6.05	3.70	4.02	0.71	6	4.49	4.03	5.08	4.09	4.89	0.38	**0.02**
	6 months	122	4.05	2.20	5.89	3.91	4.18	0.76	10	4.23	3.51	4.95	3.51	4.95	1.01	0.45
	12 months	68	3.82	2.15	4.96	3.68	3.97	0.60	6	4.62	2.32	5.72	3.11	6.14	1.45	0.16
HDL cholesterol [mmol/l]	3 months	79	1.23	0.69	2.48	1.14	1.31	0.38	4	1.01	0.80	1.29	0.68	1.34	0.21	0.24
	6 months	118	1.15	0.48	2.80	1.09	1.22	0.36	13	1.08	0.67	1.40	0.97	1.19	0.19	0.75
	12 months	68	1.04	0.58	1.79	0.98	1.10	0.24	7	0.88	0.60	1.21	0.68	1.07	0.21	0.09
LDL cholesterol [mmol/l]	3 months	76	2.12	0.89	4.40	1.99	2.24	0.55	8	2.41	1.22	3.22	1.81	3.01	0.72	0.12
	6 months	121	2.33	0.88	4.09	2.22	2.45	0.66	11	2.35	1.16	3.59	1.89	2.82	0.69	0.81
	12 months	73	2.27	1.14	3.37	2.15	2.39	0.51	5	3.21	1.73	4.92	1.73	4.68	1.19	**0.045**
Triglycerides [mmol/l]	3 months	73	1.96	0.66	6.32	1.72	2.21	1.05	8	2.17	1.03	3.72	1.33	3.00	1.00	0.45
	6 months	117	2.01	0.44	6.59	1.78	2.23	1.25	13	2.32	0.53	5.71	1.41	3.23	1.50	0.52
	12 months	63	1.56	0.44	3.87	1.37	1.75	0.76	11	1.93	0.65	3.69	1.18	2.69	1.12	0.42
ApoA1 [g/l]	3 months	65	1.41	0.20	2.18	1.34	1.48	0.28	6	1.35	1.12	1.55	1.19	1.52	0.16	0.61
	6 months	110	1.36	0.95	2.09	1.32	1.40	0.22	13	1.32	1.08	1.56	1.24	1.41	0.14	0.69
	12 months	68	1.20	0.20	1.78	1.15	1.25	0.22	7	1.05	0.85	1.33	0.89	1.22	0.18	**0.046**
ApoB [g/l]	3 months	63	0.76	0.20	1.32	0.72	0.81	0.18	12	0.80	0.51	1.06	0.68	0.92	0.19	0.58
	6 months	113	0.85	0.33	1.52	0.82	0.89	0.21	13	0.84	0.44	1.17	0.72	0.96	0.19	0.99
	12 months	68	0.79	0.45	1.14	0.75	0.82	0.16	7	0.97	0.58	1.59	0.67	1.28	0.33	0.11

The reference values for serum lipids during pregnancy [1] were used to divide between "normal" and "dyslipidemia". Dyslipidemia in maternal lipid concentrations was defined as: total cholesterol ≥ 8.99 mmol/l, HDL cholesterol ≤ 2.90 mmol/l, LDL cholesterol ≥ 6.13 mmol/l, Triglycerides ≥ 3.75 mmol/l, ApoA1 ≤ 1.65 g/l or ApoB ≥ 1.94 g/l

The Mann–Whitney *U* test for independent samples was used to test for significant differences between the groups. A *p* value < 0.05 was assumed as significance. Significant associations are highlighted in bold

*N* number, *min* minimum, *max* maximum, *95% CI* 95% confidence interval, *SD* standard deviation, *p* *p* value, *HDL* High-Density Lipoprotein, *LDL* Low-Density Lipoprotein

For the maternal HDL cholesterol concentration in the 36th week of gestation, an inverse relation to children’s height, weight, head circumference and BMI was observed, which was consistently traced up to the U6 (Table 3). From the U7, this correlation could no longer be shown. Up to the U3 examination, there was also an inverse relation between children’s anthropometry and maternal ApoA1 concentration. The inverse influence of the maternal HDL concentration was also shown for the infant’s chest (*r* = – 0.11; *p* = 0.004) and abdominal circumference (*r* = – 0.08; *p* = 0.036) at the age of 3 months. At 6 months, this association could no longer be shown. On the other hand, a higher maternal triglyceride concentration in the third trimester seems to correlate with a significantly greater infantile abdominal and chest circumference up to the age of 6 months. Maternal non-HDL cholesterol levels in the third trimester correlated with birth weight (*p* = 0.004) and neonatal BMI (*p* < 0.001) (Table 3). After adjusting for maternal BMI or maternal BMI and SES by multivariate regression analysis, the mentioned relations were no longer detectable. The influence of maternal lipid concentrations in the second trimester on infant anthropometry is shown in Supplement, Table S5.

### Serum lipids

The maternal serum lipid concentrations for the total collective as well as divided for the second and third trimesters are listed in Table 1. The mean children’s serum lipid levels at the ages of 3, 6 and 12 months can be found in Supplement, Table S4. For the total collective, the maternal BMI correlated with the maternal HDL cholesterol concentration (*r* = – 0.10; *p* < 0.001), the triglycerides (*r* = 0.29; *p* < 0.001), ApoB (*r* = 0.14; *p* = 0.003) and the non-HDL levels (*r* = 0.08, *p* = 0.003). There was no significant relation between the maternal BMI in the second (Supplement, Table S5) or third trimester (Table 3) and the lipid concentrations within the first year of children’s life. The results of the correlations between maternal and infant lipid concentrations for the second and third trimesters are summarized in Table S5 and Table 3. A higher maternal total cholesterol concentration at the 36th week of gestation seems to correlate with significantly higher total cholesterol values in children at the age of 3 as well as 6 months. A similar association could be found for the mother–child HDL (*p* = 0.015), LDL (*p* = 0.049) and ApoA1 (*p* = 0.026) concentrations in the 3rd month of life (Table 3). No significant relation could be found between infant blood pressure values and children lipid concentrations in the first year of life, respectively, the maternal lipid concentrations in the third trimester.

Taking into account the reference values for serum lipids of pregnant women [1] and the assumption of dyslipidemia ≤ 5th or ≥ 95th percentile, 49 mothers had hypercholesterolemia (≥ 8.99 mmol/l), 61 had too low HDL cholesterol concentrations (≤ 1.34 mmol/l), 32 too high LDL cholesterol levels (≥ 6.13 mmol/l), 100 a hypertriglyceridemia (≥ 3.75 mmol/l), 23 too low ApoA1 concentrations (≤ 1.65 g/l) and 28 too high ApoB levels (≥ 1.94 g/l). The lipid concentrations of the children at the respective ages of 3, 6 and 12 months, depending on the presence of a maternal normo- or dyslipidemia, are presented in Table 4. Due to the small number of cases, the results between normo- versus dyslipidemia differ not statistically significant, but the mean values in almost all groups show clear differences in concentration and thus, indicate a trend. The child's total cholesterol concentration at the age of 3 months differs significantly, despite the small number of cases (normolipidemia 3.86 ± 0.71 versus hypercholesterinemia 4.49 ± 0.38; *p* = 0.02) (Table 4). When the maternal lipid levels were differentiated into normal- and dyslipidemia and BMI values were compared into these subgroups, only in triglycerides a significant difference was found (*p* = 0.014).

### Delivery mode

A total of 75.8% (*n* = 699) vaginal deliveries, 17.5% (*n* = 166) cesarean sections and 6.7% (*n* = 62) vaginal-operative (forceps, vacuum extraction) deliveries were observed. The distribution of delivery modes depending on the fetal position is shown in Supplement (Table S2). Each subscript letter indicates a subset of delivery mode categories whose column proportions are not significantly different at the 0.05 level. Depending on the position of the fetus, there were significant differences between spontaneous delivery and cesarean section (*p* < 0.001). The delivery mode did not correlate with the maternal BMI (*p* = 0.375) or the socioeconomic status measured by Winkler Index (*p* = 0.435).

The rate of premature birth, defined as delivery < 37.0 weeks of gestation, was 5.7% (*n* = 54). One child was born in the 28.6 wog. There was no significant correlation between preterm birth and SES (mean Winkler score premature birth 14.8 ± 3.2 versus mature birth 14.6 ± 3.3; *p* = 0.545).

## Discussion

We found, that a higher maternal BMI was associated with a significantly lower Winkler score and a higher infant weight, height, head circumference and BMI from birth up to the 4th–5th week of life. In addition, the Winkler Index correlates with maternal HDL cholesterol concentration in the third trimester, which was inversely related to children’s height, weight, head circumference and BMI up to the first year of life. The results show first in an exemplary manner the complex, epigenetic influences on childhood lipid concentrations and growth in a German mother–child cohort.

Birth weight and child development are influenced multifactorial [16–18], among others by the lipid concentrations of the mother during pregnancy.

The relation between LDL- as well as total cholesterol on birth weight, described by Okala et al., could not be found here [17]. The determination of Okala's maternal lipids was carried out in the 20th and 30th weeks of pregnancy. This lack of relation may be attributed to the much earlier determination time compared to the LIFE-Child study, since the knowledge that the serum lipids show strong physiological concentration changes in the course of pregnancy [1]. Thus, comparability is difficult. Likewise, Wang et al., who also carried out the determination of the lipids in the 24th and 36th week of pregnancy, this investigation showed an inverse correlation between maternal HDL concentration and birth weight of the newborns [16], whereby the HDL concentration also seems to influence growth and weight in the longer term up to the first year of life. The influence of maternal BMI on birth weight [19] and the correlation to SES [20] has already been shown in other studies. However, no study could be found that examined the influence of maternal BMI or serum lipid levels on anthropometric markers other than birth height or weight, as shown here.

Just like infant anthropometry, the serum lipid concentrations of newborns are influenced multifactorial [21–24]. Among other things, the lipid concentrations of the mothers seem to be related to the lipid concentrations of the children (Table 3), although this connection could no longer be demonstrated after adjusting for maternal BMI and/or SES. The lipid concentrations of the children shown in Table 4, divided by maternal normo- and dyslipidemia, showed no statistical significance, but there is a trend: children of dyslipidemic mothers showed a clearly disadvantageous lipid profile with regard to cardiovascular risk. “Cardiometabolic dysfunction during pregnancy may not only contribute to long-term effects of the mother and child's vascular health but also potentially create cardiovascular risk for generational offspring.” [25]. In addition, non-HDL cholesterol is considered a cardiovascular risk factor. Higher non-HDL levels in childhood correlate with an increased carotid intima-media thickness in adulthood [26]. Our results show, that maternal non-HDL cholesterol levels in the third-trimester correlate with maternal BMI as well as birth weight and neonatal BMI. A correlation between non-HDL and the SES could not be found. All these results illustrate the importance of a healthy lifestyle with normal lipid levels during pregnancy.

Of course, a large number of additional factors not examined here also influence the lipid concentrations of children within the first few months of life, such as the influence of breastfeeding [22] or nicotine consumption during pregnancy [27]. The latter was deliberately neglected in this study, since the smoking status of the mother during pregnancy was not recorded when the LIFE-Child Study started recruiting, and the number of complete mother–child pairs would have been even lower. The influence of breast-feeding and nutritional habits of the mothers on the lipid concentrations of newborns and the relation to their SES is currently being carried out in another sub-analysis of the LIFE-Child study.

The considerable number of 982 mother–child pairs with regular follow-up examinations is a major advantage of this study. In addition, this is the first study ever to investigate the relationship between maternal lipid concentrations during pregnancy and the various parameters of child anthropometry as well as the socioeconomic status in a German cohort.

It is critical to note that the LIFE Child study design includes a single blood measurement per visit. Thus, short-term intra-individual variation of blood lipids due to biological variation and preanalytical and analytical errors may have influenced our results. The use of standardized procedures (fasting subjects, standardized time of taking blood, standardized analysis protocols) carried out by trained professionals should minimize those effects as far as possible within the framework of the LIFE Child study. Due to the invasive blood sampling to determine serum lipids, many parents also refuse the blood test in the first year of life, so that the subgroup analyzed comprised a significantly small number of complete mother–child pairs. Due to the small number of cases, it is much more difficult to show statically significant differences. Nevertheless, the results identified at least clinically relevant trends. In addition, it must be critically noted that the study population of LIFE-Child is not (completely) representative of a Caucasian cohort. Families with a low SES are clearly underrepresented at 3.6% compared to the middle and upper classes. Moreover, pregnancy-related pathologies are underrepresented. The analysis of the pregnancy cohort of the LIFE Child Study showed a prevalence of gestational diabetes of 2.7%, pregnancy-associated hypertension of 0.7% and a rate of the premature birth of 6.4% [1]. The rate of preterm birth (≤ 37.0 weeks of gestation) in the current mother–child cohort was only 5.7% versus around 8% in Saxony/Germany [28] and 13% in the perinatal center of the University Hospital Leipzig (UKL). The relation, found in other studies, between maternal dyslipidemia and preterm birth [29] could not be shown in our results, which may be due to the low prevalence. Interestingly, the generally well-documented positive correlation between maternal BMI and frequency of cesarean section could not be shown in this mother–child cohort [30, 31]. However, it is questionable whether this is only due to the low prevalence of 17.5%. There are a few studies that were also not able to show this connection [32]. Since the LIFE-Child cohort is a primarily healthy collective with few pathologies and since the majority of the probands were delivered at the perinatal center of University Hospital Leipzig, which already has a lower caesarean section rate of around 25% (including high-grade multiple pregnancies, extreme premature births (≤ 28.0 wog), preeclampsia, HELLP syndrome, placenta previa or abnormal invasive placentas), compared to the national average of around 30% [33], it can be assumed that there is indeed no relation between the maternal BMI and the C-section rate in this cohort. The positive preselection of the LIFE Child cohort and the resulting low rate of preterm birth, although participation in the study does not provide for any exclusion criteria, may result from the greater health awareness in families with middle and higher socioeconomic status [20] and therefore the greater willingness to participate in studies.

Nevertheless, this does not diminish the results of this study. Rather, it can be assumed that if more families with a lower SES participated, the effects would be even more pronounced. The results of this mother–child analysis show an inverse relationship between maternal BMI and the Winkler Index. A similar association has also been described for childhood [20, 34]. Likewise, a high SES was associated with higher maternal serum lipid concentrations of HDL cholesterol and ApoA1, which are considered cardioprotective. As already shown in preliminary studies, a higher SES in childhood also correlates with a cardioprotective lipid profile [3], although this connection could not be shown in the present study collective, due to the small number of lipid determinations in the first year of life. Therefore, it can be assumed that the differences would be even more pronounced if the SES would uniformly distribute over the study collective. It is important to recognize that children with low SES are at disadvantage in terms of cardiovascular health and the mother's social background during pregnancy effect this consistently. Therefore, preferably pregnant women and children with a low SES should be given priority in prevention programs to compensate for health disadvantages.

## Conclusion

Serum lipids and anthropometric measurements such as birth weight, abdominal girth or BMI in the first year of life are influenced multifactorial, not least essentially by maternal serum lipid levels and the maternal body mass index, while these maternal factors correlate with socioeconomic status. This knowledge is important with regard to the prevention of cardiovascular risk factors from an early age and illustrates the need for a healthy lifestyle even during pregnancy. Children from pregnant women with low socioeconomic status are particularly disadvantaged.

## Data Availability

The data that support the findings of this study are not openly available due to reasons of sensitivity, especially child data and are available from the corresponding author upon reasonable request.

## Abbreviations

SES: Socioeconomic status, measured by Winkler Index
HDL: High-density lipoprotein
LDL: Low-density lipoprotein
SDS: Standard Deviation Score
Wog: Weeks of gestation
BMI: Body-Mass-Index
KiGGS: Studie zur Gesundheit von Kindern und Jugendlichen in Deutschland
